# Realizing the Clinical Potential of Immunogenic Cell Death in Cancer Chemotherapy and Radiotherapy

**DOI:** 10.3390/ijms20040959

**Published:** 2019-02-22

**Authors:** Bernardo L. Rapoport, Ronald Anderson

**Affiliations:** 1 Department of Immunology, Faculty of Health Sciences, University of Pretoria, Pretoria 0001, South Africa; ronald.anderson@up.ac.za; 2 The Medical Oncology Centre of Rosebank, Johannesburg 2196, South Africa; 3 Institute for Cellular and Molecular Medicine, Faculty of Health Sciences, University of Pretoria, Pretoria 0001, South Africa

**Keywords:** calreticulin, damage-associated molecular patterns (DAMPs), dendritic cells, high mobility group box1, immunogenic cell death, immune checkpoint inhibitors, monoclonal antibodies, type I interferons

## Abstract

Immunogenic cell death (ICD), which is triggered by exposure of tumor cells to a limited range of anticancer drugs, radiotherapy, and photodynamic therapy, represents a recent innovation in the revitalized and burgeoning field of oncoimmunnotherapy. ICD results in the cellular redistribution and extracellular release of damage-associated molecular patterns (DAMPs), which have the potential to activate and restore tumor-targeted immune responses. Although a convincing body of evidence exists with respect to the antitumor efficacy of ICD in various experimental systems, especially murine models of experimental anticancer immunotherapy, evidence for the existence of ICD in the clinical setting is less compelling. Following overviews of hallmark developments, which have sparked the revival of interest in the field of oncoimmunotherapy, types of tumor cell death and the various DAMPs most prominently involved in the activation of antitumor immune responses, the remainder of this review is focused on strategies which may potentiate ICD in the clinical setting. These include identification of tumor- and host-related factors predictive of the efficacy of ICD, the clinical utility of combinatorial immunotherapeutic strategies, novel small molecule inducers of ICD, novel and repurposed small molecule immunostimulants, as well as the critical requirement for validated biomarkers in predicting the efficacy of ICD.

## 1. Introduction

Prior to the past decade, it had been widely believed that the human immune system was largely ineffective in protecting against the development and spread of cancer. This was attributed to various mechanisms, including lack of immunogenicity of spontaneous human tumors and, more likely, their propensity to suppress antitumor host defenses. Early evidence in support of the latter mechanism was derived from the clinical utility of interleukin (IL)-2, approved by the Food and Drug Administration (FDA) of the USA for the treatment of metastatic renal cell carcinoma and metastatic melanoma in 1992 and 1998, respectively. Additional evidence was derived from the observation that the combination of adoptive transfer of in-vitro-expanded tumor infiltrating lymphocytes/high-dose IL-2 immunotherapy demonstrated efficacy in the treatment of refractory metastatic melanoma [1].

The key turning point, however, in the now thriving field of oncoimmunotherapy was unquestionably the discovery of inhibitory immune checkpoint (IICP) molecules expressed on antitumor T lymphocytes and their counterligands on tumor cells, enabling tumors to evade immune recognition and elimination. The first of these to be discovered were programmed cell death protein 1 (PD-1) and cytotoxic T lymphocyte-associated antigen 4 (CTLA-4) by Tasuku Honjo and James P. Allison in 1992 and 1996, respectively [2,3]. In recognition of these seminal discoveries, these eminent biomedical scientists were jointly awarded the 2018 Nobel Prize for Physiology or Medicine. 

Importantly, the discovery of these and other IICP molecules, together with major advances in the genetic engineering of monoclonal antibodies (Mabs), has resulted in the development of a series of IICP-targeted Mabs, many of which have been approved for clinical use. The first of these, ipilimumab, a fully human IgG1 Mab, reactive with CTLA-4, was approved by the FDA in 2011 for the treatment of advanced melanoma. Several of these have subsequently been approved for clinical application, while others are undergoing early-stage clinical evaluation [4]. Currently, ipilimumab (an anti-CTLA4 antibody), the anti-PD1 antibodies, pembrolizumab and nivolumab, as well as the anti-PDL1 antibodies, atezolizumab, durvalumab, and avelumab have been approved for clinical application, while tremelimumab (an anti-CTLA4 antibody) is undergoing early-stage clinical evaluation [4,5].

Other types of emerging or proven oncoimmunotherapeutic strategies, which, like those based on the neutralization of IICP molecules, can be administered either as monoimmunotherapy or in combination, include but are not limited to chimeric antigen receptor (CAR) T cell therapy, therapeutic dendritic cell vaccines and, more recently, induction of immunogenic cell death (ICD). The latter type of immunotherapy, which has seemingly been practiced inadvertently for several decades, is currently limited to a group of anticancer chemotherapeutic agents, radiation therapy, and photodynamic therapy [6,7,8]. Although its therapeutic potential remains to be fully realized, ICD undoubtedly holds considerable promise and represents the focus of the current review, which prioritizes and updates the following topics:

- Definition of ICD together with a brief consideration of other types of cell death resulting from cancer therapy;
- Induction of ICD in cancer by chemotherapy and radiation therapy;
- The major types and key involvement of damage-associated molecular patterns (DAMPs) in triggering ICD;
- Evidence implicating the existence, involvement, and therapeutic benefit of ICD in the clinical setting of cancer chemotherapy and radiotherapy;
- Host defense- and tumor-related factors, which influence the antitumor efficacy of ICD;
- Combinatorial strategies based on ICD together with other types of oncoimmunotherapies;
- Novel inducers of ICD, as well as putative pharmacological adjuvant strategies that may augment ICD;
- Identification of predictive biomarkers, preferably systemic, but also in situ, which can be used to monitor the persistence and therapeutic efficacy of ICD.

## 2. Immunogenic Cell Death (ICD)

Unlike classical apoptosis, which is essentially immunoquiescent, ICD, also known as immunogenic apoptotic cell death or immunogenic apoptosis, results from exposure to diverse agents of chemical, physical or infective origin (reviewed in Reference [6]), which trigger both intracellular stress mediated by reactive oxygen species (ROS) and structural alterations to the endoplasmic reticulum (ER), leading to the sequential release of DAMPs, which, in turn, initiate antitumor immune responses [9,10]. Although ER stress-driven intrinsic apoptosis appears to represent the predominant mechanism of ICD, it is noteworthy that other modes of ICD have also been described, including necroptosis and pyroptosis [11,12]. Accordingly, and in keeping with the recommendations of the Nomenclature Committee on Cell Death 2018, ICD has been defined as a “form of regulated cell death (RCD) that is sufficient to activate an adaptive immune response in immunocompetent hosts” [13].

Building on the foundations laid by several earlier provocative studies [14,15,16], Casares et al. in 2005 were apparently the first to demonstrate that the anthracycline anticancer agent, doxorubicin, induced ICD in various murine models of experimental tumorigenesis both in vivo and ex vivo [17]. In these studies, intratumoral inoculation of doxorubicin into established or excised tumors in immunocompetent mice was found to result in tumor regression, which was prevented by depletion of dendritic cells (DCs) or CD8^+^ cytotoxic T cells [17]. This convincing demonstration of the contribution of ICD to tumor eradication, which was confirmed in a plethora of subsequent experimental studies, clearly challenged existing dogma (at that time) that the therapeutic effects of anticancer drugs are mediated solely via cytotoxicity. These findings were quickly followed by the demonstration of the critical involvement of the ER-derived DAMP, calreticulin (CRT), in the induction of ICD triggered by exposure of tumor cells to chemotherapeutic agents of the anthracycline class [18], and subsequently by the involvement of other types of DAMPs in ICD.

### 2.1. Types of Cell Death Relevant to Anticancer Treatment

As mentioned above, types of cell death commonly occurring in cancer cells during cancer therapy include mostly intrinsic apoptosis, and, less commonly, extrinsic apoptosis or necroptosis (reviewed in Reference [13]). These are summarized as follows.

#### 2.1.1. Intrinsic Apoptosis

Intrinsic apoptosis is a form of RCD activated by various microenvironmental stressors, including growth factor withdrawal, DNA damage, ER stress, reactive oxygen species (ROS) and calcium overload, replication stress, microtubular alterations or mitotic defects, which induce mitochondrial dysfunction [19,20,21,22,23,24,25].

**Figure 1:**
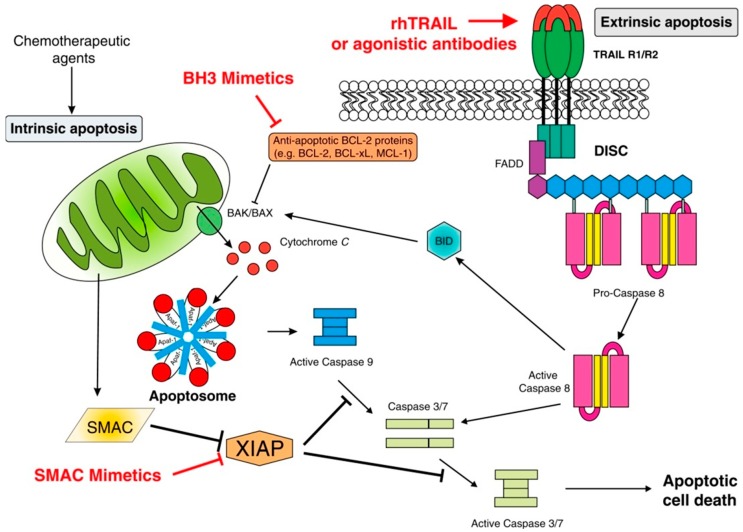
Intrinsic and extrinsic apoptotic signalling pathways and points of therapeutic intervention. Apoptosis can be initiated by signals originating from either the plasma membrane via death receptor ligation (extrinsic pathway) or at the mitochondria (intrinsic pathway). Stimulation of the extrinsic pathway by tumor necrosis factor (TNF)-related apoptosis-inducing ligand (TRAIL) results in TRAIL receptor (TRAIL-R) aggregation and formation of the death-inducing signaling complex (DISC), in which pro-caspase 8 becomes activated and initiates apoptosis by direct cleavage of downstream effector caspases. The addition of either agonistic TRAIL-R1/R2 antibodies or recombinant human TRAIL (rhTRAIL) has been used to trigger the extrinsic pathway for therapy. The intrinsic pathway is regulated by the B cell lymphoma (BCL)-2 family of proteins, which regulate pore formation in the outer mitochondrial membrane and release of apoptogenic factors, such as cytochrome c or second mitochondria-derived activator of caspase (SMAC) from the mitochondria. The release of cytochrome c into the cytosol triggers caspase-9 activation through the formation of the cytochrome c/Apaf-1/caspase-9-containing apoptosome complex. SMAC promotes caspase activation through neutralising the inhibitory effect of integrin-associated proteins (IAPs). The intrinsic pathway has been targeted for therapy either by blocking the inhibitory action of the pro-survival BCL-2 family proteins with BH3 mimetics or by inhibiting the anti-apoptotic action of IAPs with SMAC mimetics. The extrinsic and intrinsic pathways are interconnected, for example, by BID, a BH3 domain-containing protein of the BCL-2 family, which, upon cleavage by caspase-8, triggers intrinsic apoptosis, thereby further amplifying the signal from the extrinsic pathway. Reproduced with the approval of the authors: Fox; MacFarlane. “Targeting cell death signalling in cancer; minimising “Collateral damage”.” *Br. J. Cancer.* **2016**, *115*, 5–11, doi:10.1038/bjc.2016.111. Review. PMID: 27140313. Licensed under the Creative Commons Attribution 4.0 Inernational License (http://creativecommons.org/licenses/by/4.0/) (Reference [24] in the text).

The intrinsic pathway of apoptosis is controlled by the B cell lymphoma (BCL)-2 family of regulator proteins [26]. In this context, pro-apoptotic stimuli initiate upregulation of Bcl-2 homology-3 (BH3)-only protein (pro-apoptotic Bcl-2 protein containing only the BH3 domain). These proteins then activate the mitochondrial pore-forming proteins, BCL-associated X (BAX) and BAK (Backup) [27,28], which oligomerize, inducing mitochondrial outer membrane permeabilization (MOMP), the most significant event in the intrinsic apoptotic pathway [29]. This, in turn, results in the release of mitochondrial membrane proteins including cytochrome c, second mitochondria-derived activator of caspase (SMAC) and Omi/HtrA2 (a mitochondrial serine protease released into the cytosol during apoptosis process), which antagonizes inhibitors of apoptosis, thereby promoting caspase-independent cell death. The release of cytochrome c triggers formation of the apoptosome, comprising cytochrome c, apoptotic protease-activating factor-1 (APAF-1), dATP and procaspase-9, within which procaspase-9 is converted into caspase-9, followed by activation of the executioner caspases, caspase-3 and caspase-7, with resultant, extensive intracellular proteolysis and cell death [25,26,30]. Additional mechanisms contributing to cell death include inactivation of the X-linked inhibitor of apoptosis protein (XIAP) by the mitochondrial serine protease, Omi/HtrA2, while MOMP may also result in cell death due to loss of mitochondrial function even if caspases are not activated [31]. These events are depicted in Figure 1.

With respect to control of the mitochondrial pathway of apoptosis, converging mechanisms, which may limit the efficacy of ICD, include regulatory suppressor (p53, PTEN, and Rb) and oncogenic (PI3K/AKT, RAS-MAPK, and Myc) mechanisms; these act at the transcriptional and nontranscriptional levels and modulate cellular sensitivity, identify and repair stress-related damage, and control the expression and function of downstream apoptotic proteins [32].

#### 2.1.2. Extrinsic Apoptosis

Extrinsic apoptosis is a type of RCD activated by changes in the extracellular microenvironment in which cell death signals, also known as death receptors, bind to tumor necrosis factor (TNF) family death receptors [26]. These include Fas ligand (Fas-L also known as CD95), TNF-related apoptosis-inducing ligand (TRAIL), and tumor necrosis factor (TNF) [33]. Following ligand/receptor engagement, an adaptor protein is recruited to the death domain (DD) of the receptor [26,34].

The two most common adaptor proteins include Fas-associated death domain (FADD) and TNF receptor-associated death domain (TRADD) [33]. Initiator procaspase-8 and procaspase-10 then attach to the adaptor protein to form the death-inducing signaling complex (DISC) [26,34], resulting in the activation of both procaspases. These events, in turn, lead to activation of the executioner caspases, caspase-3, caspase-6, and caspase-7, which mediate extensive cleavage of intracellular proteins, including the cytoskeleton, leading to cell death. As with intrinsic apoptosis, the extrinsic pathway is also regulated by various mechanisms. In this context, DISC is regulated by the inhibitor, c-FLIP (FLICE-like inhibitory protein), an inhibitor of caspase-8 [26].

These events are also summarized in Figure 1.

#### 2.1.3. Necroptosis

Necroptosis is a type of RCD, which is activated by disruption of either extracellular or intracellular homeostasis and is initiated by various activators such as TNF-α, as well as ligands of either Toll-like receptors (TLRs) or the T cell receptor. In the case of TNF-α, binding of the cytokine to its receptor, TNFR1, results in the recruitment of TNFR1 binding protein TNFR-associated death protein (TRADD) and TNF receptor-associated factor 2 (TRAF2) and the receptor-interacting protein kinase (RIPK1). RIPK1, in turn, recruits RIPK3 to form the necrosome (also known as ripoptosome) [35]. Phosphorylation of the mixed lineage kinase domain-like pseudokinase (MLKL) by the ripoptosome drives the oligomerization of MLKL, enabling the insertion of MLKL into the plasma membrane and membranes of intracellular organelles, with resultant increased permeability [36,37]. This, in turn, generates an inflammatory phenotype with the resultant release of DAMPs and activation of immune responses and ICD [38]. 

RIPK3 can also be activated following interaction with the Toll-like receptor adaptor molecule 1 (TICAM1; best known as TRIF) either by activation of TLR3 via binding of double-stranded RNA (dsRNA) within endosomes, or by TLR4 activation by lipopolysaccharide (LPS) or various DAMPs at the plasma membrane [39]. In addition, activation of RIPK3 may also occur via interaction with the Z-DNA binding protein 1 (ZBP1), which operates as a sensor for cytosolic DNA-promoting type I interferon (IFN) synthesis, as well as NF-κB activation [40].

## 3. Role of Endoplasmic Reticulum (ER) Stress in the Induction of ICD

The ER of eukaryotic cells is involved in multiple cellular functions, including protein synthesis, folding, maturation, and transport. Importantly, the induction of ER stress is a key event in triggering ICD [18]. In this context, it is noteworthy that, as stated by Garg et al., “a peculiar characteristic of most, if not all, ICD inducers, is their ability to induce reactive oxygen species (ROS)-based/associated ER stress” [6]. This phenomenon has provided the basis for the categorization of inducers of ICD according to their mechanisms of induction of ER stress. Type I inducers are the predominant type and include anticancer drugs and radiotherapy [6]. Type I inducers mediate ER stress indirectly via targeting of cytosolic proteins and proteins involved in DNA replication, as well as interference with plasma membrane and mitochondrial membrane channels, resulting in intracellular oxidative stress and collateral damage to the ER [6,7]. In this context, it is believed that intense intracellular generation of ROS favors induction of ICD, while chronic low-level exposure to ROS is primarily procarcinogenic. Type II inducers, such as oncolytic viruses and hypericin-based photodynamic therapy, appear to target the ER directly, triggering ROS-mediated damage to this organelle by mechanisms that remain incompletely understood [6].

ER stress is characterized by the accumulation of misfolded and unfolded proteins in the lumen of the ER, causing disruption of ER homeostasis and triggering the unfolded protein response (UPR). Depending on the severity of ER stress, the UPR may either restore cellular homeostasis or trigger mostly apoptotic cell death. The molecular mechanisms underpinning these processes have been described in detail in several recent reviews [7,41]. 

**Figure 2:**
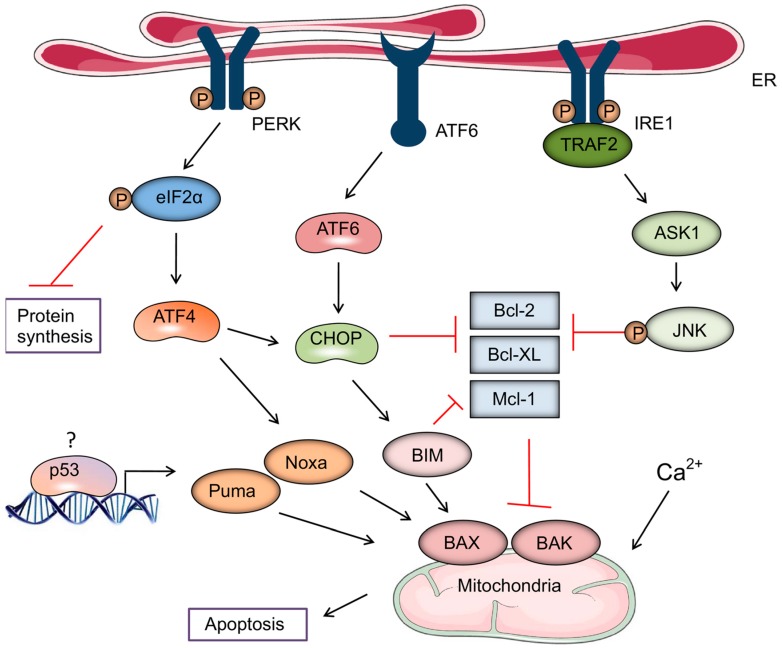
Stress-induced cell death. Under endoplasmic reticulum (ER) stress, protein kinase RNA-like endoplasmic reticulum kinase (PERK) is activated and phosphorylates and inactivates eukaryotic initiation factor 2α (eIF2α). This results in the selective induction of activating transcription 4 (ATF4) and its downstream proteins C/EBP-homologous protein (CHOP) and Noxa, resulting in cell death. CHOP, which can also be induced by ATF6, induces Bim and inhibits Bcl-2. It is still not clear how p53 is induced under ER stress and induces Noxa and Puma, resulting in apoptosis. Inositol-requiring enzyme 1 (IRE1α) can recruit TNF receptor-associated factor 2 (TRAF2), which activates apoptosis signal-regulating kinase 1 (ASK1) and its downstream target c-Jun N-terminal kinase 1 (JNK). JNK can induce apoptosis by inhibiting anti-apoptotic proteins such as Bcl-2 and Bcl-xL. Reproduced with the permission of the authors, editor-in-chief and publisher (Wiley): Iurlaro; Muñoz-Pinedo. “Cell death induced by endoplasmic reticulum stress.” *FEBS J.* **2016**, *283*, 2640–2652, doi:10.1111/febs.13598. PMID: 26587781 (Reference [41] in the text).

Briefly, ER stress results in the sequential activation of three ER stress sensor receptors viz. protein kinase RNA-like endoplasmic reticulum kinase (PERK), activating transcription factor 6 (ATF6) and inositol-requiring enzyme 1 (IRE1). Activated PERK attenuates general protein synthesis via phosphorylative inactivation of eukaryotic initiation factor 2α (eIF2α), which also results in the recruitment of activating transcription 4 (ATF4) [41]. ATF4, in turn, promotes activation of the pro-apoptotic, Bcl-2 family protein, Noxa, and the transcription factor, C/EBP-homologous protein (CHOP), which is also activated by ATF6. These events converge on BAX and BAK to initiate the mitochondrial pathway of apoptosis, which is potentiated by the activation of IRE1-driven sequential activation of apoptosis signal-regulating kinase 1 (ASK1) and c-Jun N-terminal kinase 1 (JNK), resulting in the inactivation of the anti-apoptotic proteins Bcl-2, Bcl-XL, and Mcl-1 [41]. These mechanisms are depicted in Figure 2, reproduced with permission from Iurlaro et al. [41].

These events are also linked to Ca^2+^ signaling, activation of the secretory pathway, resulting in release and translocation of DAMPs [7].

## 4. ICD in Cancer Chemotherapy and Radiation Therapy

There is evidence that the clinical benefit of conventional chemotherapy and radiation therapy is not exclusively related to direct cytotoxic effects on cancer cells but also results from the restitution of immunosurveillance. This process is characterized by cell–surface translocation of CRT as mentioned above, extracellular release of adenosine-5′-triphosphate (ATP) and high mobility group box 1 protein (HMGB1)/receptor of advanced glycation end-products (RAGE), chemokine release and stimulation of type I interferon (IFN) responses [42,43,44], the latter mediated in part via the interaction of damaged nucleic acid originating from dead and dying tumor cells with nucleic acid-sensing TLRs expressed on neighboring, viable tumor cells [42,43,44,45,46].

## 5. Types and Key Involvement of Damage-Associated Molecular Patterns (DAMPs) in Triggering ICD 

Some DAMPs, such as ATP and HMGB1, are secreted or released, while others, such as CRT and heat shock protein 90 (HSP90), are exposed de novo, or become enriched on the outer leaflet of the plasma membrane [18,47,48]. As mentioned above, the production of ROS and ER stress are essential components of the intracellular pathways that are involved in ICD, such as those involving various anticancer chemotherapeutic agents (cyclophosphamide, mitoxantrone, anthracyclines, oxaliplatin, bortezomib) and radiation therapy [8]. The combined action of ROS and ER stress triggers the activation of danger signalling pathways that help to traffic DAMPs to the extracellular space [49,50]. It is important to point out, however, that DAMPs that are released as a result of cellular stress do not always trigger an immune response. Some DAMPs, such as HGMB1, can be inactivated by oxidation, or by caspase-dependent proteolysis, as occurs with IL-33 [51,52,53].

DAMPs, such as surface-exposed CRT, secreted ATP, and passively released HMGB1, can trigger innate host defenses through interactions with various types of receptor, including the CD91 receptor on antigen-presenting cells (APCs). This interaction may enable delivery of tumor cell-derived antigens. CD91 activation also facilitates induction of APCs. HMGB1 derived from dying cancer cells may bind to TLR4 on the APC and appears to contribute to the maturation of DCs, as well as to T cell responses and production of type I interferons [54]. 

In their review, Garg et al. have provided an extensive description of DAMPs associated with cell death pathways [6], and the following are representative of some of the predominant and other DAMPS mobilized during ICD.

### 5.1. Calreticulin

CRT plays an essential role during apoptosis and, when exposed on the surface of a dying cell (ecto-CRT), serves as a phagocytic signal to DCs to trigger an innate immune response [18,55]. The presence of CRT on the cell is a potent ‘eat me’ signal and mediator of tumor immunogenicity in multiple human cancers and is critical for antitumor immunity. Additionally, the presence of ecto-CRT increases proportionally, dependent on the stage of apoptosis (pre-apoptotic, early apoptotic, and mid-to-late apoptosis). CRT is also released into the surrounding milieu and can interact with phagocytes.

CD47, also known as integrin-associated protein (IAP), is a transmembrane protein, which attenuates the prophagocytosis (‘do not eat me’ signal) function of CRT, the expression of which is increased when high levels of CRT are present on cancer cell surfaces [56].

### 5.2. Heat Shock Proteins (HSPs)

Although some controversy exists [57], it appears that heat shock proteins (HSPs), specifically HSPs 70 and 90, as well as HSPs 60 and 72 and GP96 contribute to ICD [6]. In the case of HSPs 70 and 90, these DAMPs translocate to the cell membrane during ICD, where they interact with their respective counter-receptors CD40 and CD91 on DCs [58]. Interaction of HSP70 with the co-stimulatory molecule CD40, promotes activation of CD8^+^ cytotoxic T cells [58], while the interaction of HSP90 and GP96 with CD91 on DCs contributes to cross-presentation of tumor antigens to cytotoxic T cells, possibly potentiated by TLR2 and TLR4 [59].

### 5.3. High Mobility Group Box 1

HMGB1 is a protein of 215 amino acid residues, which is organized into three distinct domains: Two tandem HMG box domains (A box and B box), which are spaced by a short flexible linker, and a 30 amino acid–long acidic C-terminal tail [60]. HMGB1 is released from dying cancer cells that are undergoing necrosis, including necroptosis (programmed necrosis). HMGB1 release is associated with an inflammatory response via interaction with several receptors, including TLR2, TLR4, TLR9, and RAGE [61].

### 5.4. Adenosine 5-triphosphate (ATP)

Extracellular release of ATP is also a a recognized hallmark of ICD, acting as a ‘find me’ signal via the attraction of immature DCs, monocytes/macrophages, and neutrophils to sites of dead and dying tumor cells by a mechanism involving interactions of ATP with P2Y2 receptors on these cells [62,63]. Additional mechanisms of ATP-mediated antitumor immunity include interaction of the nucleotide with another type of purinergic receptor, viz. the P2X7 receptor on DCs, causing activation of the NLR domain-containing protein 3 (NLRP3) inflammasome with resultant intracellular proteolytic processing of pro-IL-1β and increased secretion of the mature, pro-inflammatory cytokine [48].

### 5.5. Spliceosome-Associated Protein 130 (SAP130)

ICD, as well as necroptotic cell death, also results in the release of preformed nuclear proteins such as SAP130. This is a component of the U2 small nucleoprotein complex involved in the assembly of spliceosomes (a nuclear component comprised of small noncoding RNAs and proteins, which deletes introns from pre-mRNA) [64]. SAP130 activates innate immune responses linked to adaptive immunity via binding to a macrophage-inducible, cell surface C-type lectin known as Mincle/Clec4e/Clecsf9. This is “an activating receptor that interacts with an Fcγ receptor, which contains immunoreceptor tyrosine-based activation motifs (ITAMS) involved in mediating inflammatory responses to necrotic cells” [64]. SAP130/Mincle-mediated macrophage activation via Fcγ receptor binding triggers the production of pro-inflammatory cytokines/chemokines, which, in turn, contribute to antitumor immunity via neutrophil recruitment [64]. In addition to macrophages, Mincle is also expressed on DCs, providing a link to activation of adaptive immunity [65].

### 5.6. Defensins and S100 Proteins

These DAMPs are also released from dying tumor cells and appear to contribute to ICD via activation of inflammatory mechanisms linking innate host defenses to adaptive antitumor immunity. Defensins comprise two families of cysteine-enriched, membrane-active antimicrobial peptides viz. the α- and β-defensins. The former are located predominantly in cells of the innate immune system, mainly neutrophils, while β-defensins have a broader distribution, occurring both in cells of the immune system and epithelial cells, the latter accounting for the majority of cancers. In the case of β-defensins, specifically β-defensins 1 and 3, these peptides interact with monocyte-derived DCs, possibly through binding to TLRs 2 and 4, which in turn, transform these cells to a highly-activated phenotype associated with significantly upregulated expression of the activation marker, CD91 [66,67].

S100 proteins belong to a family consisting of at least 25 low molecular weight, Ca^2+^-binding proteins found mostly in the cytosol, but also in the nucleus and plasma membrane of many cell types, including cancer cells [68]. Several members of the S100 family, specifically S100A8, S100A9, and S100A12, are released from dying cancer cells and appear to contribute to ICD via binding to RAGE as described above for HMGB1 [6].

## 6. Effects of DAMPs Released during Cell Death on Activation of Innate and Adaptive Immune Mechanisms

The volume of evidence implicating DAMPs originating from dying cancer cells in the process of ICD is overwhelming, with the important caveat that this contention is derived almost exclusively from in vitro studies and murine models of experimental tumorigenesis. Because innate and adaptive immune mechanisms involved in the induction of ICD in experimental settings have been covered extensively in many excellent recent reviews, together with the paucity of compelling evidence in relation to the involvement of these mechanisms in the clinical setting, this section is covered briefly. 

The key mechanism triggering antitumor immune responses during ICD appears to involve recruitment and activation of APCs, most importantly DCs and macrophages, by DAMPs, with CRT, HSPs, ATP, and HMGB1 particularly effective with respect to induction of interferons α and β [6]. Notwithstanding the ability of DCs to drive T cell polarization towards an antitumor Th1 phenotype, these cells can also migrate to regional lymphoid tissue, where they may further augment antitumor T cell responses. Macrophages of the M1 phenotype, in particular, also contribute to tumor cell killing, either directly via the production of the cytokines, IL-1β and tumor necrosis factor-α (TNF-α), ROS and reactive nitrogen intermediates (RNIs), as well as indirectly via the production of the natural killer (NK)-activating cytokine, IL-12.

In addition to driving antitumor CD8^+^ cytotoxic T cell reactivity during ICD, DCs also mobilize NK cells to eradicate tumor cells, an activity which may be of particular importance in controlling metastasis [8,69]. Various subsets of DCs, both plasmacytoid and myeloid, appear to contribute to NK cell activation by various mechanisms, including secretion of IFN-α, IL-2, IL-12, IL-15, and IL-18, all of which promote NK cell cytolytic activity via various mechanisms, including: (i) Interaction of NK cells with the chemokine, CXC3L1 expressed on DCs, which triggers activation of release of IFN-γ by NK cells; (ii) activation of NKp46 and NKG2D receptors, which also triggers IFN-γ release; and (iii) production of the cytotoxic proteins, perforins, and granzymes by NK cells (reviewed in Reference [69]).

Clearly, DCs are critical drivers of ICD. This contention is underscored by the fact that targeted knockout of the gene encoding the DC chemoattractant receptor, formyl peptide receptor 1 (FPR1), abrogates ICD induced by anthracycline [70]. In this setting, ANXA1, which is normally located on the inner plasma membrane, also functions as a DAMP, translocating to the exterior of the tumor cell during apoptosis, where it functions as a counterligand for FPR1 on immature DCs [6,70]. These ANXA1/FPR1 interactions promote DC maturation and activation, eliciting a T cell-medated antitumor immune response [70]. 

Interestingly, a loss of function allele of this gene has been proposed to underpin the poor metastasis-free and overall survival in breast and colon cancer patients receiving adjuvant chemotherapy [70].

**Figure 3:**
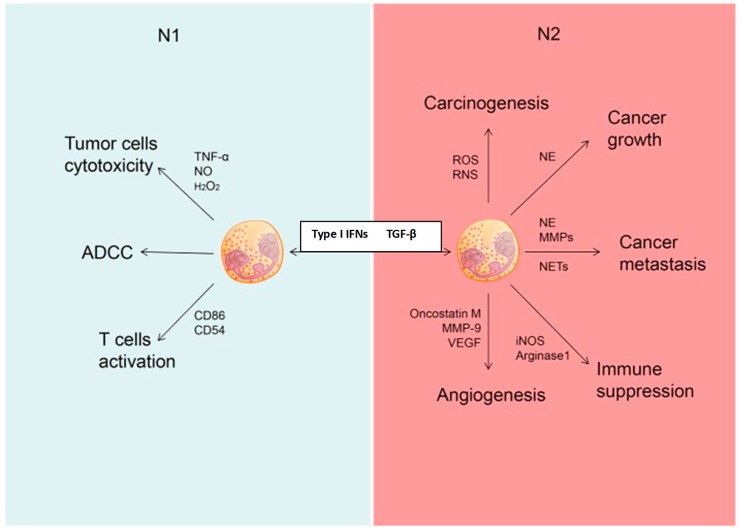
Different roles of N1 and N2 neutrophils in cancer. Neutrophils could be polarized into N1 phenotype under the induction of type I interferon (IFNs) and polarized into N2 phenotype under the induction of transforming growth factor-β1 (TGF-β1). N1 neutrophils may inhibit the development of cancer through tumor cell cytotoxicity, antibody-dependent cell-mediated cytotoxicity (ADCC), as well as activation of T cells. N2 neutrophils may promote carcinogenesis, tumor growth, metastasis, and angiogenesis, as well as immunosuppression. N0: Nitric oxide; H_2_O_2_: Hydrogen peroxide; CD86 and CD 54: Co-stimulatory molecules; ROS: Reactive oxygen species; RNS: Reactive nitrogen species; MMP: Matrix metalloproteinase; VEGF: Vascular endothelial growth factor; NE: Neutrophil elastase; NETs: Neutrophil extracellular traps; iNOS: Inducible nitric oxide synthase. Reproduced with the approval of the authors with minor modifications: Wang; Qiu; Li; Wang; Yi. “Understanding the multifaceted role of neutrophils in cancer and autoimmune diseases.” *Front. Immunol.* **2018**, *9*, 2456, doi:10.3389/fimmu.2018.02456. PMID: 30473691 (Reference [72] in the text).

In addition to DCs and monocytes/macrophages, and probably structural cells, recent studies have identified the neutrophil as a potentially significant contributor to immunorestoration during ICD. These cells are also recruited to the tumor environment by chemokines, specifically the combination of CXCL1, CCL2, and CXCL10, released from immunogenic, dying cancer cells [71]. Though it was previously believed to be predominantly protumorigenic, this perception of the role of the neutrophil in tumor immunity has changed following the recognition of distinct neutrophil phenotypes with opposing activities. These are the N1 and N2 phenotypes, which possess antitumorigenic and protumorigenic properties, respectively [72]. Importantly, type I interferons play a key role in polarizing neutrophils towards the N1, tumor-suppressive phenotype, while transition to the N2 phenotype is mediated by transforming growth factor-β1 (TGF-β1) [72,73]. The N1 phenotype is characterized by production of the DC and cytotoxic T cell recruiting/activating cytokines/chemokines TNF-α, IL-12, CCL3, CXCL9, and CXCL10 [72,73]. Additional mechanisms of tumor immunity mediated by N1 cells include antibody-dependent cell-mediated cytotoxicity and enhancement of antigen presentation via increased expression of co-stimulatory molecules [72]. The respective, counteracting roles of N1 and N2 neutrophils in tumor immunity, are summarized in Figure 3. On a cautionary note, however, the relevance of the N1/N2 to the clinical setting of cancer therapy remains to be conclusively established [74].

## 7. Evidence in Support of the Existence and Efficacy of ICD in the Therapeutic Setting

Although immunohistochemistry based on the detection of tumor-infiltrating lymphocytes (TILs) and regulatory T cells (Tregs) has shown prognostic utility in monitoring the clinical efficacy of IICP-based immunotherapy, demonstration of a clear and unambiguous association between induction and therapeutic efficacy of ICD in the clinical setting has proved to be more difficult, as mentioned above. This is a reflection of a number of factors, including but not limited to: (i) The limited range of agents, which induce ICD in the clinical setting, together with the paucity of clinical studies which have addressed this issue; (ii) lack of knowledge in relation to the best combinations, dosages, frequencies, and duration of administration of ICD-inducing agents; (iii) difficulty in monitoring efficacy due to restrictions on the performance of follow-up biopsies; (iv) very importantly, the current lack of appropriate systemic biomarkers in particular; (v) the vast range of malignancies and stages of disease; and (vi) apparent induction of ICD according to immunohistological detection of associated biomarkers in the absence of clinical response [75,76,77,78].

Nevertheless, one such study has reported encouraging findings. In this context, Ladoire and colleagues recently reported on histological analysis of biomarkers of ICD, specifically HMG-B1 and LC3B^+^, STQSM1/p62 (the latter two are biomarkers of active autophagy) present on biopsies taken from 1798 patients with breast cancer [79]. These authors reported significant associations between loss of HMGB1 expression and active autophagy with a poor prognosis [79]. On the other hand, Aoto et al., in a study focused on detection of biomarkers of ICD, specifically CRT and HMGB1, in pretreatment biopsy specimens and surgically resected specimens taken from either patients with breast cancer (*n* = 52) or esophageal squamous cell carcinoma (ESCC, *n* = 8), who had been treated with neo-adjuvant chemotherapy (NAC), reported less convincing findings [77]. These authors found that although administration of NAC to patients with both types of malignancy resulted in significantly increased expression of both CRT and HMGB1 relative to pretreatment levels, these changes in expression of the two DAMPs did not correlate with responses to either NAC or patient survival. The authors concluded that although chemotherapy alone can induce ICD in patients with breast cancer and ESCC, “that combination chemotherapy of CRT or chemotherapy with immune checkpoint inhibitors may therefore induce a synergistic effect” [77]. 

In this latter context, Garg et al. reported in late 2017 that at least 58 clinical trials are currently focused on induction of ICD by anticancer chemotherapeutics in various types of malignancy. Twenty of these involve agents, such as doxorubicin, epirubicin, bleomycin, oxaliplatin, and bortezomib, as well as the combination of idarubicin with mitoxantrone; all of these agents are being used in combination with various other chemotherapeutic and immunotherapeutic strategies [80]. The remaining trials are based on cyclophosphamide, mostly in combination with other ICD inducers, IICP Mabs, DC vaccines, or recombinant DAMPs [80]. With respect to induction of ICD by radiation therapy, Walle et al. reported in early 2018 that more than ninety clinical trials assessing the effects of the combination of radiotherapy and immunotherapy are ongoing, with over 40 of these evaluating the clinical efficacy of radiotherapy in combination with PD-1-targeted monoclonal antibodies [81,82].

## 8. Properties of Tumors and Host Defenses that Determine the Efficacy of ICD

Notwithstanding the potential of only a restricted range of chemotherapeutic and other agents to induce ICD, the most significant predictors of antitumor efficacy are clearly related to the tumor genotype/phenotype and efficacy of antitumor host defenses. Weak tumor immunogenicity, the efficacy of host antitumor defences, and the intensity of tumor-associated immunosuppression therefore represent the major barriers which must be overcome by ICD. In this context, ICD may counteract both host- and tumor-related immunosuppression.

### 8.1. Tumor-Related Factors Impacting on the Efficacy of ICD

Many types of cancer, such as glioblastoma and ovarian cancer, often have a low mutational load and are consequently poorly immunogenic due to low rates of antigenicity [83]. Others, such as pancreatic ductal cancer, appear to be particularly adept at creating highly immunosuppressive tumor microenvironments [84]. Melanomas and nonsmall cell lung cancer (NSCLC), on the other hand, are among the more highly immunogenic tumors, which are often more responsive to oncoimmunotherapy [85]. However, even in this setting, the efficacy of ICD and other types of cancer immunotherapy may be compromised by tumor-mediated immunosuppression. Several of these mechanisms, excluding the expression of IICP molecules on infiltrating cytotoxic T cells, are considered in the following sections.

#### 8.1.1. Tumor Mutational Burden

The importance of the tumor mutational burden as an independent predictor of both tumor immunogenicity and response to immunotherapy has recently been highlighted by Greil et al. [86]. Even more recently, Lyu et al. devised a mutation load estimation model based on only twenty-four genes as a predictor of the response to IICP Mab cancer immunotherapy [87]. These authors investigated patients with lung adenocarcinoma using a computational framework based on the “somatic mutation data downloaded from The Cancer Genome Atlas (TCGA) database” [87]. The authors reported that the estimated mutation load enabled identification of patients with “durable clinical benefits”, the sensitivity, specificity, and accuracy values being 85%, 93%, and 89%, respectively. Although necessitating more extensive evaluation in the clinical setting, this type of tumor mutational modeling may be extrapolatable to other types of cancer and is possibly more affordable than other procedures, such as those based on whole exome sequencing [87].

#### 8.1.2. Tumor Expression of PD-L1

Expression of PD-L1 is a strategy commonly used by tumor cells to engage PD-1 on T cells, thereby suppressing antitumor immunity. Upregulation of tumor cell expression of PD-L1 has, however, been reported following exposure of melanoma and glioblastoma cells to combination chemoradiation in vitro [88], as well as in the clinical setting during treatment of patients with cisplatin or chemoradiation for head and neck squamous cell carcinoma or rectal cancer, respectively [89,90]. In this context, and as advocated by the authors of all three of the aforementioned studies [88,89,90], adjunctive Mab-mediated blockade of PD-L1, or other checkpoint inhibitors, represents an adjunctive immunotherapeutic strategy to augment the efficacy of ICD [91]. This contention is supported by the findings of a recent experimental murine melanoma study, which demonstrated the synergistic immunorestorative effects of gamma-irradiation in combination with PD-1-targeted monoclonal antibodies [92], which is in keeping with the immune stimulatory potential of radiotherapy and possibly hypofractionated irradiation in particular [93]. Interestingly, anti-PD-L1 Mabs have also been demonstrated to be effective in murine models of experimental cancer chemotherapy against non-PD-L1-expressing tumors [91]. The mechanism underpinning this alternative mechanism of anti-PD-L1-directed immunotherapy appears to involve neutralization of PD-L1 expressed on antigen-presenting cells, resulting not only in restoration of T cell activation, but also in the recruitment of these cells to sites of tumor invasion [91].

#### 8.1.3. Overexpression of CD47 and MHC Class I

As with expression of PD-L1 on antigen-presenting cells, especially DCs, expression of CD47, already mentioned above, on cells of the innate immune system also impedes the efficacy of ICD and other types of immunotherapy. CD47 belongs to the immunoglobulin superfamily [94]. It is a transmembrane protein of molecular weight 50 kDa that binds to various ligands, such as thrombospondin and signal regulatory protein α (SIRPα) [94]. Interaction of CD47 expressed on tumor cells with SIRPα expressed on DCs and other phagocytic cells of the innate immune system effectively counteracts the “eat me” signals delivered by surface CRT and HSP90 [94].

More recently, a second ‘do not eat me’ signaling mechanism has been identified on tumor cells, which is mediated via the expression of MHC class I molecules [95]. In this setting, MHC class I, via interactions with the leukocyte immunoglobulin-like receptor B1 (LILRB1) expressed on macrophages, suppresses the phagocytic activity of these cells. However, the involvement of this mechanism in regulating the efficacy of ICD remains to be established.

#### 8.1.4. Immunosuppressive Factors Released by Tumor Cells

Tumor cells also release a range of immunosuppressive agents with potent immunosuppressive activities which may counteract ICD. For example, the cytokines IL-10 and TGF-β1 promote the induction and differentiation of Tregs, while suppressing DC maturation and inducing the transition of macrophages to the quiescent M2 phenotype, which is associated with reduced expression of the key ICD-inducing receptor, FPR1 [83,96,97]. Other major immunosuppressive factors overexpressed by tumor cells include the following:

- Adenosine: While extracellular ATP is considered to be a major effector of ICD, it is also a substrate for ectonucleotidase enzymes, most prominently CD39 and CD73 [98]. These enzymes are highly expressed on various cell types present in the tumor microenvironment, including tumor cells per se, structural cells, and cells of both the innate and adaptive immune systems, particularly Tregs [98]. These ectonucleotidases, in turn, convert ATP to adenosine, a potent endogenous, immunosuppressive agent. Interaction of adenosine with adenosine 2A (A2_A_) subtype receptors, which are linked to activation of the enzyme, adenylyl cyclase, results in the synthesis of broadly immunosuppressive 3’,5’-cyclic adenosine monophosphate (cAMP) [98];
- Prostaglandin E_2_ (PGE_2_): It is well-recognized that many different types of cancer (colorectal, prostate, lung, pancreatic, breast) overexpress the enzyme, inducible cyclooxygenase 2 (COX-2), resulting in excessive production of PGE_2_ [99]. Operating via EP2 and EP4 receptors, PGE_2_, like adenosine, causes activation of adenylyl cyclase and synthesis of cAMP [99];
- Indole-2,3-dioxygenase: This enzyme is expressed by both tumor cells and cells of the innate and adaptive immune systems. It metabolizes tryptophan to kynurenine, anthranilic acid, and 3-hydroxyanthranilic acid, all of which are immunosuppressive and contribute to the evasion of immune-mediated eradication of tumors [100,101].

These various tumor-associated mechanisms, which may impede the efficacy of ICD and other oncotherapeutic strategies, are summarized in Table 1.

### 8.2. Host-Associated Factors Which May Restrict the Efficacy of Immunogenic Cell Death

#### 8.2.1. Chronic Infections

Chronic infections with hepatitis B virus (HBV) and hepatitis C virus (HCV) are a global health problem, with 500 million people chronically affected. Factors associated with reduced immune response include viral escape mutations leading to lack of recognition by antiviral immune cells and loss of antiviral effector functions of virus-specific CD8+ T cells, called T-cell exhaustion [102]. In addition, the hallmark of human immunodeficiency virus (HIV) infection is a progressive depletion of CD4^+^ T-cell populations, resulting, amongst others, in predisposition for development opportunistic infections and various types of malignancy [103].

#### 8.2.2. Smoking

Cigarette smoking is associated with acquired immune dysfunction affecting both adaptive (T cells) and innate (DCs, macrophages, NK cells, epithelial cells) immune mechanisms, as well as a pro-iflammatory response characterized by increased numbers and reactivity of circulating and pulmonary neutrophils [104,105]. While immune dysfunction is likely to be mediated in part by the suppressive activities of toxicants present in cigarette smoke, it also appears to be related to the production of immunosuppressive ROS and arginase generated by smoke-activated neutrophils [105]. In this context, an association between persistent inflammation, immunosuppression, and cancer is well recognized [106].

#### 8.2.3. The Inflammatory Tumor Microenvironment

Malignant cells are surrounded by an infiltrate consisting of stromal fibroblasts and bone marrow-derived cells, comprising macrophages, neutrophils, lymphocytes (B and T), and NK cells. These immune cells release a variety of soluble mediators, including cytokines and chemokines, which contribute to maintaining an inflammatory, protumorigenic microenvironment [107].

#### 8.2.4. Immunosenescence

Immunosenescence is a process that refers to the gradual deterioration of the immune system associated with aging. Age-related, acquired immune dysfunction of both the innate and adaptive immune systems results in increased susceptibility to infectious diseases, cancer, and autoimmune pathologies. Production of inflammatory mediators is a crucial feature of aging, as well as being a crucial component of the tumor microenvironment, as mentioned earlier [107]. 

#### 8.2.5. Obesity, Co-Morbidities, and Mental and Physical Stress

In general, chronic nonresolving inflammation increases the risk of cancer. Tumor promotion is also augmented by systemic conditions associated with ongoing inflammation, such as chronic co-morbidities, chronic stress, and obesity [107].

These various factors that may impact negatively on antitumor host defenses are summarized in Table 2.

## 9. Strategies to Augment the Efficacy of ICD in Cancer Chemotherapy and Radiotherapy

At least three major strategies aimed at potentiating the clinical efficacy of ICD have been identified. Firstly, as mentioned earlier and expanded on below, the therapeutic efficacy of ICD is likely to be improved considerably through the pretherapy implementation of procedures that enable detection of the level of tumor immunogenicity, as well as the immunocompetence of patients, to identify those who are most likely to benefit most from the induction of tumor ICD; secondly, and perhaps most promisingly, combining ICD with alternative immunotherapies, particularly Mab-mediated neutralization of IICP molecules as discussed below; thirdly, and somewhat more speculatively at this stage, the development of novel, pharmacological inducers of ICD, which augment the current, very limited group of ICD-inducing chemotherapeutic agents, as well as those which counteract tumor-mediated immunosuppression.

### 9.1. Pretherapy Selection of Patients

Although not yet in common practice, the following criteria may enable detection of those most likely to experience and benefit from ICD:

- Type of malignancy;
- Normal circulating lymphocyte count and neutrophil:lymphocyte ratio, together with low numbers of immunosuppressive neutrophils;
- Low levels of circulating immunosuppressive cytokines, specifically IL-1Ra, IL-2R, IL-10, and TGF-β1;
- High tumor mutational burden, which may be performed at reasonable cost in the future [87];
- Digital image analysis of tumor bopsies to detect and enumerate types of infiltrating antitumor effector cells and suppressor cells; one such procedure gaining prominence is the “Immunoscore” [108];
- Presence of PD-L1 and/or CD47 on tumor cells and/or antigen-presenting cells, which may identify those patients likely to benefit from PD-1- and/or CD47-targeted immunotherapy; although extensive work has been done on PD-L1, CD47 remains to be validated in this context;
- Expression of IICP molecules on T cells in tumor biopsies, or the presence of systemic, soluble forms of these molecules;
- Specific epigenetic profiling termed “EPIMMUN”, which is based on the establishment of DNA methylation microarrays [109]. This strategy, albeit preliminary, has shown promise in identifying those patients with NSCLC who are likely to benefit from anti-PD-1 Mab therapy [109]. Together with the composition of the tumor immune cellular infiltrate, detection of the unmethylated status of the Treg transcription factor, forkhead box P1, was found to predict clinical response [109]. However, this strategy requires more intensive clinical evaluation and evidence of affordability.

These various strategies, which may assist in identifying those cancer patients who are most likely to benefit from immunotherapy, are summarized in Table 3.

### 9.2. Combinatorial Immunotherapeutic Strategies

Although immunotherapy represents a major breakthrough in the management of patients with malignant disease, only a quarter-to-a-third of the patients benefit long term. To achieve greater therapeutic success, combination therapies are required, including but not limited to radiation therapy, chemotherapy, monoclonal antibodies, also including those which augment positive immune checkpoint molecules (e.g., OX40 and CD137), cancer vaccines, and anti-angiogenesis agents. Currently, combinatorial therapies are being investigated in most types of human malignancy. These have been highlighted in several very recent reviews and are not discussed further here. In this context, however, we do foresee a growing need for the development of additional, reliable biomarkers of immunorestoration and tumor elimination to enable a more rationale approach to combinatorial therapy.

### 9.3. Small Molecule-Based Immunopharmacological Strategies

In the context of this review, these are essentially of two types: Firstly, those specifically focused on ICD and, secondly, those which may augment tumor immunity by mechanisms other than induction of ICD.

#### 9.3.1. Repurposed Agents Which Act as Inducers/Enhancers of ICD

Several categories of pharmacological agents, which are not recognized as standard cancer chemotherapeutic agents, have been reported either to induce or to act as adjuvants of ICD. However, these remain largely untested in the setting of clinical oncoimmunotherapy and include the following:

#### 9.3.2. Cardiac Glycosides

This class of ICD-inducing agents includes those such as digoxin, which are used widely in the treatment of atrial fibrillation and heart failure via inhibition of the Na^+^/K^+^-ATPase pump [110]. In addition, several other types of cardiac glycosides, including those within the National Cancer Institute (NCI) mechanistic diversity set, have also been identified as effective inducers of ICD during screening procedures using various cancer cell lines [110,111]. Interestingly, retrospective clinical analyses revealed that patients who were receiving digoxin during chemotherapy for breast, colorectal, head and neck, or hepatocellular cancer with anticancer agents other than ICD-inducing anthracyclines or oxaliplatin experienced improved overall survival, possibly attributable to ICD [112,113].

Although it seems unlikely that these agents will gain prominence as clinically useful inducers of ICD due to issues of cardiotoxicity, it is noteworthy that the cationic amphiphilic antibiotic, clofazimine, is also a potent inhibitor of eukaryotic cell Na^+^, K^+^-ATPase, and P-glycoprotein-mediated drug efflux, effects which are secondary to membrane destabilization [114]. Although apparently untested as an inducer of ICD, clofazimine also possesses other mechanisms which may contribute to ICD, such as pro-oxidative potentiation of the eradication of tumor cells by phagocytes [115].

##### Nonsteroidal Anti-Inflammatory Drugs (NSAIDs)

Although these agents are generally thought to augment antitumor immune reactivity via inhibition of the synthesis of broadly immunosuppressive PGE_2_ by tumor cells, a very recent study has also implicated their involvement in the induction of ICD [116]. The authors reported “a critical role of ER stress upstream of death receptor signaling and BID activation” (BID is the BH3 only domain Bcl-2 pro-apoptotic protein), which was associated with the appearance of markers of ICD and DC activation both in colorectal cell lines and colorectal-cancer susceptible APC^Min/+^ mice [116]. In addition, the authors also mention the presence of “elevated levels of ER stress and cell death markers in advanced adenomas from patients taking NSAIDs including aspirin” [116]. 

#### 9.3.3. Novel Small Molecule Inducers of ICD

##### Septacidin

The same screening strategy of the NCI mechanistic diversity set also identified an N-acyl-amino acid antibiotic with known antitumor activity viz. septacidin as an inducer of ICD both in tumor cell lines in vitro, as well as in murine models of experimental oncoimmunotherapy [110].

##### LTX-315

This is also a cationic amphiphilic, membrane-disruptive agent, which is a chemically modified 9-mer peptide, which mimics the structure of naturally-occurring antimicrobial peptides [117]. Based on its activity both in vitro and in experimental animal models, LTX-315 appears to fulfil all the criteria of an effective ICD-inducing agent [117]. In addition, LTX-315 per se has demonstrated efficacy as an intratumorally administered treatment for solid tumors [117] and has recently completed phase I/II evaluation in combination with IICP Mabs as an intratumoral treatment for various types of advanced solid malignancies [118].

##### CBP501

This agent is a calmodulin-binding peptide, which promotes uptake of platinum into cancer cells [119]. In experimental settings, both in vitro and in vivo, it has been demonstrated to promote the transition of cisplatin from an ineffective to an effective inducer of ICD [119]. It is currently being evaluated in combination with cisplatin and nivolumab (PD-1 inhibitor) in a phase Ib clinical study involving patients with advanced refractory tumors [120].

##### Dinaciclib

This agent is a cyclin-dependent kinase inhibitor, which effectively induces ICD in exposed tumor cell lines, while demonstrating antitumor efficacy in murine models of experimental oncoimmunotherapy when used in combination with anti-PD-1 Mabs [121].

##### RT53

This agent is also a cationic amphiphilic, membrane-disruptive peptide, which effectively induces ICD in a melanoma cell line, while augmenting protective antitumor immune responses following immunization of mice with RT53-treated fibrosarcoma cells [122]. Like its counterpart, LTX-315 [117,118], local intratumoral injection of RT53 into solid tumors in mice was found to activate an immune response which resulted in tumor regression [122].

These more recently described ICD-inducing agents, many of them novel, are summarized in Table 4.

## 10. Small Molecule Immunostimulants for Cancer Immunotherapy Which Apparently Do Not Directly Induce ICD 

### 10.1. Novel Agents

A number of promising small molecules with immunostimulatory potential are currently in the preclinical and early stages of clinical evaluation. These are likely to be used in combination with other types of immunotherapy, including ICD and IICP-Mab-based strategies. Many of these novel agents, some of which have reached early phase clinical evaluation, have been very recently and comprehensively reviewed by Cheng and colleagues [123] and are summarized briefly here:

- CA-170: This is an inhibitory molecule, which targets PD-L1, as well as another IICP viz. VISTA (V-domain Ig suppressor of T cell activation [124];
- LYC-55716: This molecule is an agonist of the retinoic acid-related orphan receptor-γt (RORγt), a transcription factor intimately involved in promoting the proliferation, survival, and function of Th17 cells [125];
- Galunisertib: This agent is a potent antagonist of the tyrosine kinase domain of the TGF-β receptor type I with considerable immunotherapeutic potential [126];
- Indoximod: This agent is a prototype inhibitor of indoleamine-2,3-dioxygenase, which, together with several other similar agents, is currently under early clinical evaluation in various types of malignancy [123,127]; although a recent study reported lack of efficacy in melanoma [128], efficacy in other types of malignancy remains to be established;
- CB-1158: This agent is an inhibitor of arginase, an enzyme produced predominantly by myeloid suppressor cells, which mediates immunosuppressive activity via depletion of arginine [129];
- CPI-444 and AZD4635: These are small molecule antagonists of the T cell adenosine A2a receptor, both of which are undergoing preclinical and early stage clinical evaluation [130,131,132].

Many other small molecules with immunotherapeutic potential are currently under preclinical development, including inhibitors of the ATP ectonucleotidases CD39/CD73 [123].

### 10.2. Propranolol as a Repurposed Immunotherapeutic Agent for Cancer

Although, to our knowledge, the noncardioselective, β-adrenergic agent, propranolol, does not possess ICD-inducing activity, it does, however, antagonize adenylyl cyclase-linked type 2 β-adrenoreceptors, which are expressed on cells of both the innate and adaptive immune systems [133]. This, in turn, results in interference with synthesis of immunosuppressive intracellular cAMP, representing a broad-spectrum immunostimulatory mechanism, which has generated recent interest in the antitumor potential of propranolol. 

In this context, administration of propranolol was found to augment antitumor immunity in a murine spontaneous model of melanoma, which was associated with attenuated influx of myeloid suppressor cells and increased tumor infiltration of cytotoxic T cells [134]. Not surprisingly, the mechanism of this beneficial, antitumor effect of propranolol has been reported to result from reduction of adrenergic stress and norepinephrine-driven β-adrenoreceptor signaling [135]. From a clinical perspective, it is noteworthy that patients with metastatic melanoma treated with the combination of anti-PD-1/high-dose IL-2 immunotherapy, who were also receiving concurrent administration of propranolol, experienced improved overall survival relative to those patients who did not receive β-blocker therapy [136].

The aforementioned, low-molecular weight, pharmacological immunostimulants are summarized in Table 5. Clearly, their exact roles, as well as that of propranolol, if any, in potentiating and/or sustaining ICD in the clinical setting, remain to be established. In this context, future research should focus on the potential of these agents to perpetuate ICD during the intervals between cycles of administration of standard chemotherapeutic agents and/or radiotherapy. Additonally the sequence (concurrent as opposed to sequential treatment), dosing, and duration of treatment remain to be established.

## 11. Systemic Biomarkers Predictive of the Efficacy of ICD

Identification of reliable, reproducible, clinically validated systemic biomarkers of ICD as an adjunct or alternative to immunohistochemistry is the topic of much ongoing research, but is complicated both by lack of specificity of the considerable range of tumor cell-derived DAMPs as well as biomarkers of immunorestoration. Although Kepp et al. have formulated consensus guidelines for the detection of ICD, these are mostly applicable to the screening of novel agents with putative ICD-inducing activity [137]. Clearly, therefore, carefully-controlled studies which prioritize extended immunological monitoring are a prerequisite with respect to identifying reliable, predictive, and prognostic biomarkers, either individually, or, most likely in combination, in the clinical setting of ICD. As mentioned above, this has, however, proven to be difficult and appears to underscore the necessity for studies focused on a specific malignancy and single type of ICD-inducing therapy, incorporating serial measurement of a broad range of tumor- and immune system-derived biomarkers.

In this context, an ongoing clinical trial entitled “Detection of Circulating Biomarkers of Immunogenic Cell Death (ICD)” initiated in April 2017 by Dr. De Ruysscher and colleagues at Maastro Clinic, the Netherlands, with a projected completion date of January 2019, is noteworthy [138,139]. The study cohort consists of patients with NSCLC. Patients with stage III disease receive concurrent cisplatin–doublet chemotherapy and fractionated, non-ablative radiotherapy, while those with stage 1 disease receive stereotactic, ablative chemotherapy only. The duration of the study is 5 weeks, with systemic biomarker analysis performed on three occasions. The profile of biomarkers analyzed by these investigators is extensive and includes various DAMPs, autoantibodies to CRT and HSP90, indoleamine-2,3-dioxygenase, protumorigenic and antitumorigenic chemokines/cytokines, as well as serum-associated exosomes, the latter representing a recent innovation in the detection of exosomal, cancer-associated microRNAs [140]. This type of approach may eventually enable the identification and prioritization of systemic biomarkers representative of clinically meaningful ICD.

## 12. Conclusions

DAMPs such as calreticulin, secreted ATP, RAGE, and HMGB1 are danger signals for the immune system, which trigger the induction of ICD. Considerable experimental and laboratory advances have been made over the past decade in characterizing the DAMPs associated with ICD. Numerous agents have the potential to augment ICD in patients undergoing anticancer radiation therapy, chemotherapy or immunotherapy. However, further clinical and biomarker studies are required to establish the exact role, the timing, the doses, and the duration of treatment of ICD-inducing agents in the treatment of cancer.

## Figures and Tables

**Table 1:** Tumor-associated mechanisms which may restrict the efficacy of immunogenic cell death.

Mechanism	Consequence
Low mutational load [86,87] *	Decreased immunogenicity and immune evasion
Expression of PD-L1 on tumor cells, as well as on tumor-infiltrating macrophages [91]	Decreased immunogenicity and immune evasion
Upregulation of expression of CD47 on tumor cells [94]	Interacts with SIRPα on dendritic cells to suppress “eat me” signals
Expression of MHC class I molecules by tumor cells [95]	Interact with LILRB1 on macrophages, suppressing phagocytic activity
Release of various types of immunosuppressive factor by tumor cells [83,96,97,98,99,100,101]	IL-10 and TGF-β1 Adenosine Prostaglandin E2 Indole-2,3-dioxygenase

* Denotes the relevant references as cited in the text.

**Table 2:** Host-associated factors which may restrict the efficacy of immunogenic cell death.

Chronic infections [102,103] * Smoking [104,105] Inflammatory tumor microenvironment [107] Immunosenescence, obesity, co-morbidities, physical and mental stress [107] Others

* Denotes the relevant references as cited in the text.

**Table 3:** Strategies which may assist in identifying those cancer patients who are most likely to benefit from immunotherapy.

Strategy	Comment
Type of malignancy, e.g., NSCLC, melanoma [83,84,85] *	Tend to have higher levels of immunogenicity
Absence of lymphopenia and high neutrophil:lymphocyte ratios	Nonspecific indicators of poor antitumor immune responses
Measurement of systemic immunosuppressive cytokines [83,96,97]	IL-1Ra, IL-2R, IL-10, and TGF-β1 are of particular significance in this context
Measurement of tumor mutational load [86,87]	A high mutational load is predictive of good immunogenicity
Computerized image analysis of tumor biopsies [108]	Enables immunological profiling of the tumor microenvironment
Detection of the presence of PD-L1 and/or CD47 and MHC 1 on tumor cells [91,94,95]	Mab-targeting of these immunosuppressive proteins may promote immune eradication
Specific epigenetic profiling (“EPIMMUN”) to predict responsiveness to PD-1-targeted MAbs in NSCLC [109]	Based on detection of the unmethylated status of the Treg transcription factor, Forkhead box P1

* Denotes the relevant references as cited in the text.

**Table 4:** Novel and “re-purposed” inducers of immunogenic cell death.

Novel	Re-Purposed
Septacidin: an N-acyl-amino acid antibiotic [110] *	Cardiac glycosides: Inhibit Na^+^/K^+^-ATPase [110,111,112,113] Clofazimine: The prototype riminophenazine, cationic amphiphilic agent with membrane-disruptive activity, but untested in induction of ICD [114,115] Nonsteroidal anti-inflammatory drugs: Induce endoplasmic reticulum stress associated with death receptor signaling and BID activation [116]
LTX-315: a cationic amphiphilic, membrane-disruptive, chemically-modified 9-mer peptide [117,118]	
CBP501: a calmodulin-binding peptide that promotes uptake of cisplatin into tumor cells [119,120]	
Dinaciclib: a cyclin-dependent kinase inhibitor [121]	
RT53: a cationic amphiphilic, membrane-disruptive peptide [122]	

* Denotes the relevant references as cited in the text.

**Table 5:** Novel, low-molecular weight, pharmacological immunostimulants which may augment immunogenic cell death.

Agent	Immunostimulatory Mechanism
CA-170	Targets PD-L1 and VISTA [124] *
LYC-55716	Agonist of RORγt [125]
Galunisertib	Antagonist of the tyrosine kinase domain of TGF-β receptor type 1 [126]
Indoximod	Prototype inhibitor of indoleamine-2,3-dioxygenase [123,127,128]
CB-1158	Granulocyte arginase inhibitor [129]
CPI-444 and AZD4635	Antagonists of the T cell A2a receptor [130,131,132]

* Denotes the relevant references as cited in the text.
